# What has zinc transporter 8 autoimmunity taught us about type 1 diabetes?

**DOI:** 10.1007/s00125-019-04975-x

**Published:** 2019-08-23

**Authors:** Claire L. Williams, Anna E. Long

**Affiliations:** Translational Health Sciences, Bristol Medical School, University of Bristol, Level 2, Learning and Research, Southmead Hospital, Bristol, BS10 5NB UK

**Keywords:** Autoimmunity, B and T cells, Biomarker, Epitope specificity, Method development, Pathogenesis, Prediction, Review, SLC30A8, Type 1 diabetes, ZnT8 autoantibodies

## Abstract

Zinc transporter 8 (ZnT8), a protein highly specific to pancreatic insulin-producing beta cells, is vital for the biosynthesis and secretion of insulin. ZnT8 autoantibodies (ZnT8A) are among the most recently discovered and least-characterised islet autoantibodies. In combination with autoantibodies to several other islet antigens, including insulin, ZnT8A help predict risk of future type 1 diabetes. Often, ZnT8A appear later in the pathogenic process leading to type 1 diabetes, suggesting that the antigen is recognised as part of the spreading, rather than the initial, autoimmune response. The development of autoantibodies to different forms of ZnT8 depends on the genotype of an individual for a polymorphic ZnT8 residue. This genetic variant is associated with susceptibility to type 2 but not type 1 diabetes. Levels of ZnT8A often fall rapidly after diagnosis while other islet autoantibodies can persist for many years. In this review, we consider the contribution made by ZnT8 to our understanding of type 1 diabetes over the past decade and what remains to be investigated in future research.

**Electronic supplementary material:**

The online version of this article (10.1007/s00125-019-04975-x) contains a slideset of the figures for download, which is available to authorised users.

## Introduction

Islet autoantibodies develop before the clinical onset of type 1 diabetes and are currently the principal biomarkers for identifying individuals at risk of the disease. Detection of multiple autoantibody positivity in childhood implies an 80% risk of developing diabetes within 15 years, although the rate of progressions varies from a few months to decades [1]. In 2007, zinc transporter 8 (ZnT8) autoantibodies (ZnT8A) were characterised [2] and have since been recognised [1, 3] as one of the four major islet autoantibodies, together with GAD65 autoantibodies (GADA) [4], islet antigen-2 autoantibodies (IA-2A) [5] and insulin autoantibodies (IAA) [6].

ZnT8 is encoded by *SLC30A8* and the first genome-wide association study (GWAS) linking a polymorphism in this gene, on chromosome 8q24.11, with type 2 diabetes was published in 2007 [7]. The common single nucleotide polymorphism (SNP) in *SLC30A8*, rs13266634 (C/T), causes a non-synonymous R325W modification in the protein sequence [7, 8]. The *SLC30A8* genotype appears to impact insulin production [9] and rare protective loss-of-function mutations in this gene can increase insulin secretory capacity and reduce type 2 diabetes risk considerably [10, 11]. Numerous studies and a meta-analysis have shown that the rs13266634 SNP is not associated with overall type 1 diabetes risk in childhood [12]. However, data from the German prospective cohort studies BABYDIAB and BABYDIET suggested that *SLC30A8* may influence age at onset of diabetes and rate of progression within predisposed individuals, perhaps by its effect on insulin production [13, 14]. In this review, we consider questions concerning the autoimmune response to ZnT8 in type 1 diabetes that remain to be answered (summarised in Table 1).

**Table 1:** A summary of important unanswered questions about the immune response to ZnT8 in type 1 diabetes

Stage	Field of study		
	Genetics	B cell responses	T cell responses
Initiation of autoimmunity	How do HLA genotypes alter central tolerance for ZnT8?	Is the C-terminal always the target of ZnT8A?	What causes ZnT8 to become a target of autoimmunity?
Before diabetes	Do SNPs in *SLC30A8* influence the rate of progression in people at risk of type 1 diabetes?	Does epitope spreading occur across ZnT8? Are ZnT8A preferentially lost in rapid progressors or do they appear less often?	The broad T cell response to ZnT8 suggests previous epitope spreading; when does this occur?
At diagnosis	Do common SNPs modulate age of onset and what is the mechanism of this?	What is the frequency of ZnT8A in adult-onset T1D? How can it help us distinguish subtypes of diabetes?	How does HLA-A*24 present ZnT8 peptide compared with HLA-A*2?
During diabetes	Does the *SLC30A8* genotype predict long-term clinical outcomes following diagnosis?	Why are ZnT8A lost more rapidly compared with GADA or IA-2A? Does this correlate with beta cell function?	Why are ZnT8 T cell responses lost over the first few years after diagnosis? Is this because of rapid loss of antigen?

T1D, type 1 diabetes

## The structure and function of ZnT8

**Fig. 1:**
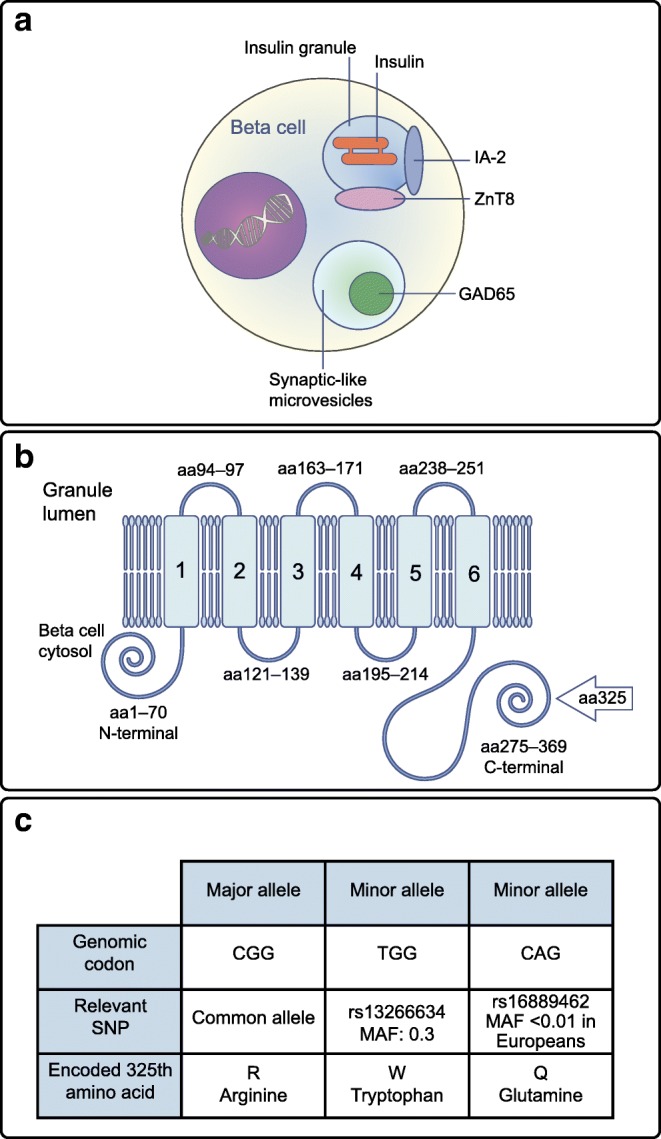
(**a**) Schematic diagram of the pancreatic islet beta cell illustrating the locations of the major antigens that autoantibodies recognise: GAD65, islet antigen-2 (IA-2), insulin and the zinc transporter ZnT8. IA-2, insulin and ZnT8 are found within insulin secretory granules but GAD65 is found within synaptic-like microvesicles within the beta cell. (**b**) The structure of the transmembrane protein ZnT8, which is embedded within insulin secretory granule membranes. The C- and N-terminals are cytosolic but the transmembrane domains (numbered 1–6) include three luminal regions, which are expressed extracellularly during insulin granule exocytosis. Adapted from [2]. Copyright 2007 National Academy of Sciences. (**c**) The codons for the major allele and the SNPs in *SLC30A8*, which encodes ZnT8. These SNPs determine protein sequence at amino acid (aa)325 in the C-terminal of ZnT8. The major allele (encoding R325) is associated with increased risk of type 2 diabetes; however, in type 1 diabetes, this SNP influences autoantibody specificity to ZnT8 and can aid risk stratification of disease progression. MAF, minor allele frequency. This figure is available as part of a downloadable slideset

A member of the zinc transporter family, ZnT8 is highly expressed in the pancreatic beta cells, where it is the most abundantly expressed zinc transporter [15]. Like other major autoantigens in type 1 diabetes, ZnT8 has high beta cell specificity and is localised to insulin secretory granules (Fig. 1a). ZnT8 is important for beta cell function, through its role in supplying zinc ions (Zn^2+^) for insulin storage and biosynthesis [16]. ZnT8 is a 369-amino acid polytopic transmembrane protein with cytoplasmic N- and C- terminal tails (Fig. 1b) [11, 17].

## The development of the ZnT8A immune response

The structure of ZnT8 suggests that less than half of the protein is accessible to immune surveillance and this may explain why ZnT8 is a target of autoimmune attack in type 1 diabetes. The whole protein or fragmented peptides are most likely to be accessible to the immune system upon beta cell death, although following granule exocytosis, during insulin secretion, the luminal transmembrane domains are exposed extracellularly [18].

### Initiation of ZnT8 autoimmunity

At diagnosis, IAA, IA-2A and GADA are associated with specific HLA class II alleles contained within type 1 diabetes risk genotypes [19, 20]. In contrast, ZnT8A have not shown consistent HLA class II associations [21], except with DQ6.4 in individuals with type 1 diabetes [22]. They are, however, associated with high diabetes risk (i.e. DR3/DR4) in first-degree relatives. Epitope scanning identified more putative ZnT8 epitopes for HLA-DQ2 than for -DQ8 or -DQ6.4 [23]. An intermediate binding epitope for HLA-DQ2 was predicted to bind W325 but not R325, suggesting that this may contribute to differences in central tolerance for ZnT8. However, regardless of DQ type, people with diabetes had a higher frequency of proinflammatory ZnT8-specific CD4^+^ T cells than age- and HLA-matched non-diabetic individuals [24].

The predisposing HLA class I A*24 allele was negatively associated with presence of ZnT8A at and before diagnosis when age at onset was accounted for [25, 26]. Studies of ZnT8 epitopes for CD8^+^ T cells have focused on the better-characterised HLA-A*2. There is evidence that CD8^+^ T cells recognise a range of ZnT8 peptides across the transmembrane/loop and C-terminal regions in individuals with diabetes [27–29]. This could suggest previous epitope spreading but may also reflect the technical challenge of detecting and characterising antigen-specific T cell responses ex vivo. The number and phenotype of ZnT8-specific T cells identified in people with and without diabetes appear to be similar but functional differences have been identified, such as greater ZnT8-stimulated IFN-γ secretion by isolated CD8^+^ T cells from people with diabetes [30]. In the pancreases of people who had type 1 diabetes, compared with type 2 diabetes or no diabetes, more ZnT8-specific CD8^+^ T cells were present, suggesting that ZnT8-specific lymphocytes home to the pancreas.

### ZnT8 as an autoantigen

Humoral islet autoimmunity can develop in children as young as 6 months of age with a peak incidence of seroconversion at 2–3 years and a probable second peak in puberty [1]. The first autoantibodies to develop in young children are typically IAA and/or GADA and, therefore, insulin and GAD65 are often regarded as primary targets of autoimmunity. In contrast, IA-2A and ZnT8A are rarely the only islet autoantibodies identified at primary seroconversion; data for ZnT8A are, however, more limited [2, 14]. Although ZnT8A can be detected close to initiation of autoimmunity, ZnT8A and IA-2A typically arise later in prediabetes and are more common in adolescents at diagnosis. Similarly, in mice, transfer of ZnT8 C-terminal-specific CD4^+^ T cells only caused diabetes if insulitis was present. Additionally, ZnT8-specific CD4^+^ T cells were only found in pancreas and lymph nodes of mice in advanced disease [31], supporting the observation that antigen spreading to ZnT8 is characteristic of the developing autoimmune response.

Most of the mature ZnT8A responses recognise the C-terminal of the protein and only 10% recognise the N-terminal [3]. Within the C-terminal, ZnT8A can be specific to amino acid 325 of ZnT8 (Fig. 1c) and this specificity is defined by the *SLC30A8* polymorphism rs13266634 [32]. Hence, individuals with the CC genotype (R325) rarely develop ZnT8 tryptophan-specific autoantibodies (ZnT8WA) and individuals with the TT genotype (W325) rarely develop ZnT8 arginine-specific autoantibodies (ZnT8RA). Competitive displacement experiments show that ZnT8A are truly specific for R325 or W325 [33]. Thus, individuals respond to endogenous ZnT8 protein determined by their own genome. This has not been as easy to demonstrate for the other islet antigens because they lack an amino acid polymorphism with such an obvious influence on the autoantibody response. In several populations, ZnT8A appear to cross-react with a viral protein from *Mycobacterium avium subsp. paratuberculosis* (e.g. [34]) and around 50% of ZnT8A-positive individuals have antibodies recognising epitopes independent of amino acid 325 [32]. Therefore, molecular mimicry could be a contributing factor in the initial response to ZnT8 but more work is needed to evaluate this.

For other islet autoantibodies, epitope spreading has been demonstrated to occur during progression, and identification of epitopes indicative of later stages of disease has improved the specificity of these markers [35, 36]. Investigations into ZnT8 epitope autoantibody responses have focused on samples taken close to disease onset. In addition to the well-characterised amino acid 325R/W polymorphism, a second SNP, rs16889462 (G/A) which encodes Q325, has also been identified but is present in <1% of Europeans. A conformational ZnT8A epitope (dependent on R332, E333, K336 and K340 but independent of R325) [37] was characterised by comparing human and mouse chimeric ZnT8 proteins. The importance of conformational epitopes to ZnT8A responses is also supported by the observation that linear 15 amino acid ZnT8 peptides were insufficient to displace ZnT8A [38]. If epitopes of ZnT8A associated with higher risk of diabetes could be identified, these would aid prediction but the pattern of epitope-specific responses before diagnosis has not so far proved useful for risk stratification.

## Predicting diabetes using ZnT8A

Wenzlau et al first described ZnT8A in 2007; analysis of 43 individuals at high genetic risk followed prospectively showed that ZnT8A could stratify risk in those positive for IAA, GADA or IA-2A alone [2]. Subsequently, the prospective birth-cohort study BABYDIAB also showed that ZnT8A aid prediction of disease in the presence or absence of other islet autoantibodies [14]. The much larger TrialNet dataset confirmed these findings and concluded that screening for ZnT8A should be included in prediction and prevention studies [39]. Several studies have shown that combined testing for IA-2A and ZnT8A identifies relatives who progress rapidly to disease in the most cost-effective way [40–42]. The contribution of ZnT8A in predicting risk is likely age-dependent; as the benefit of adding ZnT8A in single-islet-autoantibody-positive individuals in the Diabetes Autoimmunity Study in the Young (DAISY) was found when onset was after age 6 years [2]. In preselected relatives who were positive for other islet autoantibodies, ZnT8A only added to risk prediction in relatives who were older or who were at genetically lower risk [43]. Within those who progress slowly to disease, ZnT8A are frequently detected [44]. Most studies of risk stratification for type 1 diabetes have been conducted in relatives or individuals with high HLA class II risk. The added predictive value of ZnT8A in the general population has not been fully assessed but is ongoing (e.g. in the German Fr1da study [45]). Despite contradictions, current evidence suggests that screening for ZnT8A will provide additional information about risk, especially in older individuals in whom early autoimmune responses to insulin are waning.

## Lessons learnt from ZnT8A measured at diagnosis

When ZnT8A were originally characterised, these autoantibodies increased the number of people identified as single- or multiple-antibody positive at diabetes onset. Of 133 individuals negative for IAA, GADA and IA-2A at diagnosis, 26% were positive for ZnT8A and 47% of 51 people positive for a single antibody (IAA, GADA or IA-2A) became multiple-antibody positive when ZnT8A were considered [2]. Overall, several international studies have shown that around two-thirds of children are ZnT8A positive at diagnosis [32, 46]; depending on the age group considered, the prevalence is similar to IA-2A.

The importance of ZnT8A as a biomarker in adult disease has not been fully assessed. One study from Belgium suggested that only half of those diagnosed over the age of 20 years are positive for ZnT8A [40]. This frequency is less than for GADA (65%) but comparable with IA-2A (45%) [47, 48]. The contribution of ZnT8A in populations defined as LADA is difficult to determine because the characteristics of the participants vary between studies. There is, however, evidence that screening for ZnT8A reduces the cost of discriminating monogenic diabetes [49].

ZnT8A also contributed to our observation that the natural history of humoral autoimmunity may be changing. In our UK-based study, carried out between 1985 and 2002 in individuals at disease onset, the prevalence of IA-2A and ZnT8A increased while that of GADA and IAA remained stable over time [46]. This could indicate a shift towards a more aggressive form of disease, as IA-2A and ZnT8A tend to develop later in pathogenesis. During the same time period, the incidence of diabetes in younger children has increased and the proportion of probands with high genetic risk has reduced, suggesting an increase in environmental risk [50, 51]. Lower genetic risk alleles, such as DQ6.4, could therefore have become more common in people with diabetes and contributed to increased ZnT8A. A Danish study, covering a shorter time span, found no difference in prevalence of IA-2A or GADA in individuals at the time of diagnosis (1997–2005) but ZnT8A were not measured [52].

## ZnT8A as a biomarker of insulin secretory capacity post diagnosis

Another suggestion from the initial 2007 paper was that ZnT8A may be correlated with loss of insulin secretion because of ZnT8’s beta cell specificity and co-localisation with insulin [2]. Following initially successful pancreas transplant, ZnT8A have been associated with rapid onset of hyperglycaemia and eventual loss of graft function, making them a potentially important biomarker for predicting beta cell loss [53]. Ongoing beta cell death is likely to drive levels of ZnT8A [2, 54], although low-level expression in non-functional beta cells or neighbouring alpha cells may also contribute. After diagnosis ZnT8A are lost more rapidly than GADA or IA-2A [2], while T cell responses, particularly to the transmembrane domain, are also lost within a few years post diagnosis [28]. This is important because if ZnT8A, which are easier to measure than T cells, reflect insulin secretory capacity and autoreactivity, they may be useful biomarkers for assessing/monitoring the efficacy of clinical trials. In 2013, Ingemansson et al showed that in a group of 15–34 year olds with diabetes (71% type 1), high C-peptide (indicative of endogenous insulin secretion) at diagnosis correlated with prospective preservation of ZnT8A 5 years after diagnosis [55]. However, another study in individuals with younger onset showed that high positivity for ZnT8A at diagnosis was associated with lower C-peptide levels and increased insulin dose within the first 2 years after diagnosis, despite similar levels at disease onset [56]. In a minority of individuals where ZnT8A persist for decades after diagnosis, cross-sectional studies have also failed to reach a consensus about the relationship between ZnT8A and C-peptide [57–59]. These contrasting findings could be explained if age at diagnosis influences this association. The hypothesis that ZnT8A could be used as a biomarker for therapeutic effect is attractive but is not strongly supported by current literature.

## What benefits could new assays for ZnT8A offer for prediction?

**Fig. 2:**
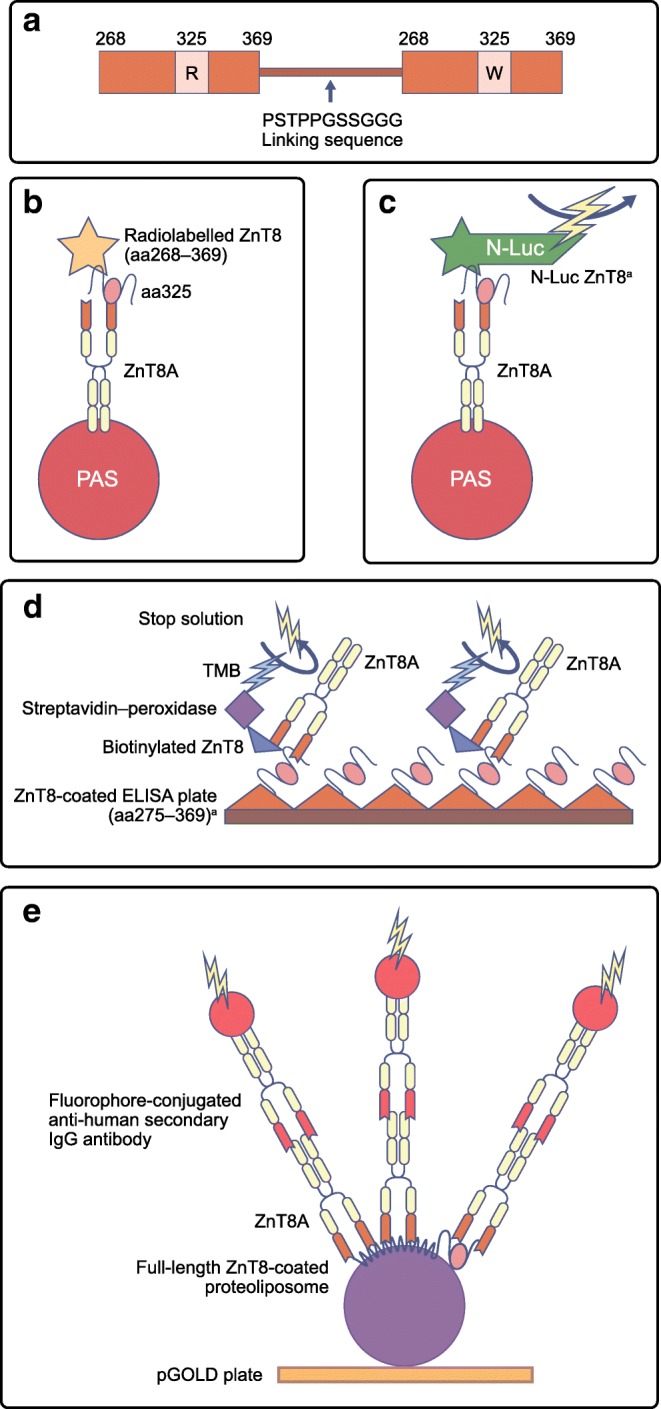
(**a**) Dimeric C-terminal probes enable measurement of ZnT8RA and ZnT8WA in a single assay [65]. Monomeric probes can also be used. (**b**–**e**) Multiple assay formats have been employed to measure ZnT8A. (**b**) The gold standard RIA, C-terminal ZnT8A assay, uses ^35^S-methionine and Protein A Sepharose (PAS) to immunoprecipitate ZnT8A in serum. (**c**) LIPS employs antigen-conjugated *N*-luciferase (N-Luc) and the substrate furimazine to produce a detectable luminescence signal. LIPS has a similar assay format to gold standard RIAs and provides an alternative to radioisotope use. (**d**) The routinely used commercial ZnT8 bridge ELISA (developed by RSR) employs plates coated in C-terminal ZnT8 and detects ZnT8A by means of a biotin–streptavidin–peroxidase system, where the peroxidase acts as a substrate to 3,3′, 5,5′-tetramethylbenzidine (TMB) to create a colourogenic reaction proportionate to the level of ZnT8A [66]. (**e**) A method for measuring ZnT8A responses outside of the C-terminal, through a three-dimensional format, has been proposed, creating scope for elucidating conformational epitopes. This method uses full-length ZnT8 and proteoliposomes immobilised on pGOLD platform plates. Detection of ZnT8A is through binding of anti-human IgG Fc secondary antibodies conjugated to a fluorophore. (**e**) Adapted from [54]. ^a^ indicates that the exact ZnT8 sequences for these assays are still being optimised (**c**) or are proprietary (**d**). aa, amino acid. This figure is available as part of a downloadable slideset

Currently, the gold standard method for measuring circulating islet autoantibodies, including ZnT8A, is RIA. Internationally, RIAs for GADA and IA-2A have been harmonised but work to standardise measurement of ZnT8A and IAA is ongoing [60]. These assays have proved highly specific and sensitive in differentiating healthy individuals from diabetic individuals and have been used to investigate C-terminal epitopes recognised by ZnT8A [2, 32]. However, this method is disadvantaged by the use of costly radioisotopes, which are tightly regulated and have limited shelf lives. The main assay development has been to create dimers (Fig. 2a) of the amino acid 268–369 sequence connected by a linking sequence to allow for the simultaneous measurement of antibodies to the most common R325 and W325 variants using RIA (Fig. 2b). In one Finnish study, addition of a Q325 probe further increased RIA sensitivity by reducing the number of antibody-negative children and adolescents [61].

Alternative assays, such as the luciferase immunoprecipitation system (LIPS) (Fig. 2c) and ELISAs (Fig. 2d), have begun matching or even exceeding the specificity and sensitivity of RIAs for GADA and IA-2A [62, 63]. While ELISAs eliminate the requirement for radioisotopes, sample volume requirements greatly exceed those of RIAs (increased from 4 μl per test to 50 μl); LIPS assays offer similar performance to RIAs with potentially even lower sample volume requirements [64]. This would facilitate the use of small volume samples such as capillary bleeds for screening young infants and the general population.

There are assays in development that may maintain the conformational structure of ZnT8. In 2017 Wan et al developed a novel assay using full-length ZnT8 (R325) in combination with proteoliposomes (Fig. 2e) [54]. This assay has the potential to detect ZnT8A that recognise epitopes outside the C-terminal but the need to use the pGOLD platform restricts routine application of this assay due to costs. Future assay adaptations to investigate other epitopes, including post-translational protein modification, and that are validated on ‘at-risk’ individuals before disease onset may improve disease prediction and should continue to be investigated.

## Conclusion

Measurement of ZnT8A is a cost-effective method by which to identify multiple-islet-autoantibody-positive individuals at increased risk of diabetes. This marker may be particularly useful in assigning risk from adolescence onwards. The study of ZnT8A has also given us vital evidence of a true autoimmune response and strengthens observations that the pathogenesis of the disease may change over generations. In the future, the influence of variants in *SLC30A8* on the rate of progression to clinical type 1 diabetes should be clarified, especially in those with age of onset above 15 years. Why autoimmunity spreads to target ZnT8 later in the pathogenesis of the disease, why this response is lost rapidly after diagnosis and whether these changes are related to beta cell function are just some of the questions that still require clarification. Analysis of ZnT8A and T cell epitopes before diagnosis will improve our understanding of disease pathogenesis and provide better biomarkers of disease.

## Electronic supplementary material

ESM (PPTX 483 kb)

## Abbreviations

GADA: GAD65 autoantibodies
IAA: Insulin autoantibodies
IA-2A: Islet antigen-2 autoantibodies
LIPS: Luciferase immunoprecipitation system
ZnT8: Zinc transporter 8
ZnT8A: ZnT8 autoantibodies
ZnT8RA: ZnT8 arginine-specific autoantibodies
ZnT8WA: ZnT8 tryptophan-specific autoantibodies
