# Supplementary material for: What has zinc transporter 8 autoimmunity taught us about type 1 diabetes?
Source: Diabetologia. 2019 Aug 23;62(11):1969–76. doi: 10.1007/s00125-019-04975-x (PMC6805822; doi:10.1007/s00125-019-04975-x)
Supplement: Supplementary file 1 — (PPTX 483 kb) [file 125_2019_4975_MOESM1_ESM.pptx]

## Slide 1
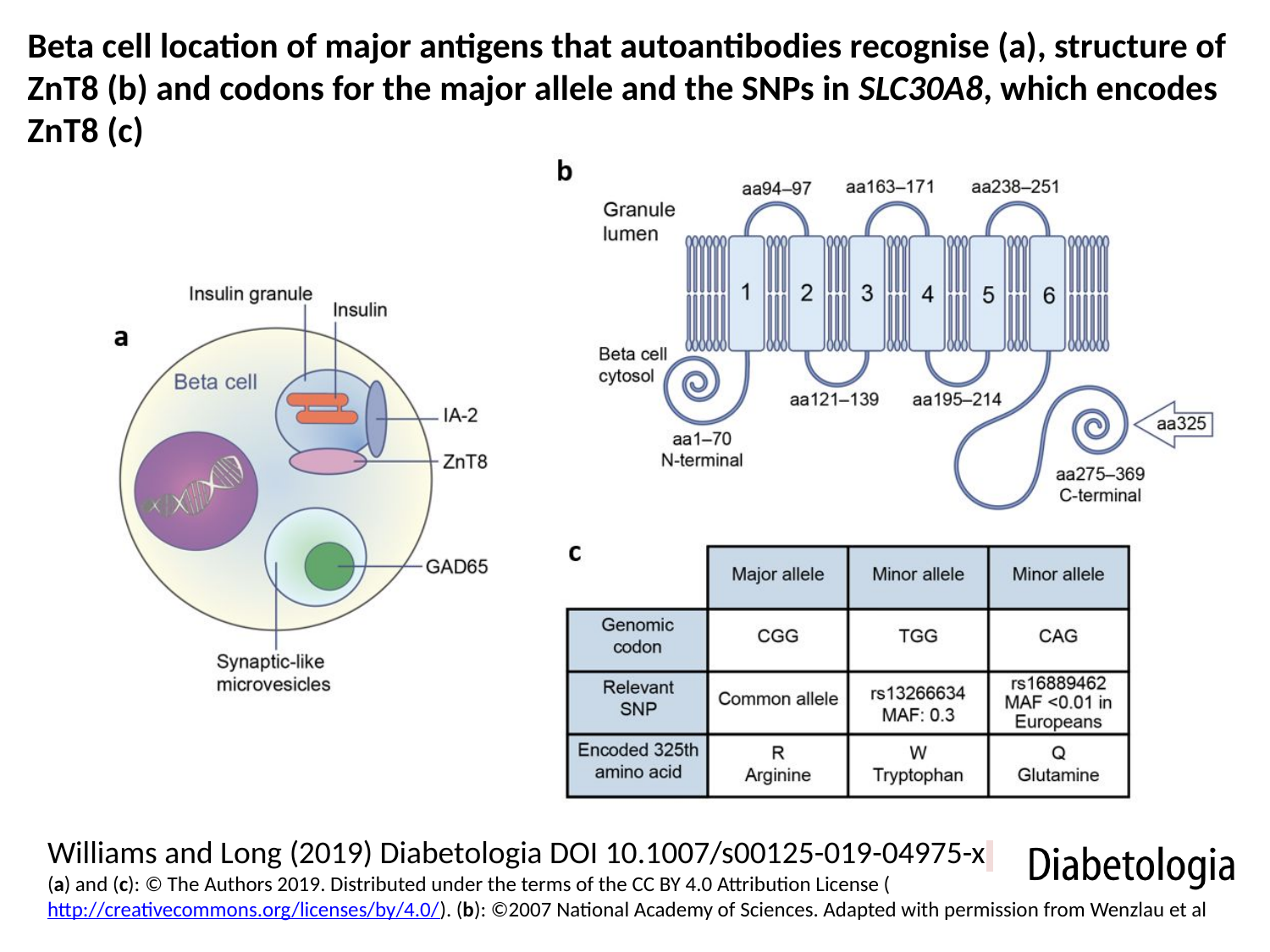

Beta cell location of major antigens that autoantibodies recognise (a), structure of ZnT8 (b) and codons for the major allele and the SNPs in SLC30A8, which encodes ZnT8 (c)
Williams and Long (2019) Diabetologia DOI 10.1007/s00125-019-04975-x
(a) and (c): © The Authors 2019. Distributed under the terms of the CC BY 4.0 Attribution License (http://creativecommons.org/licenses/by/4.0/). (b): ©2007 National Academy of Sciences. Adapted with permission from Wenzlau et al

## Slide 2
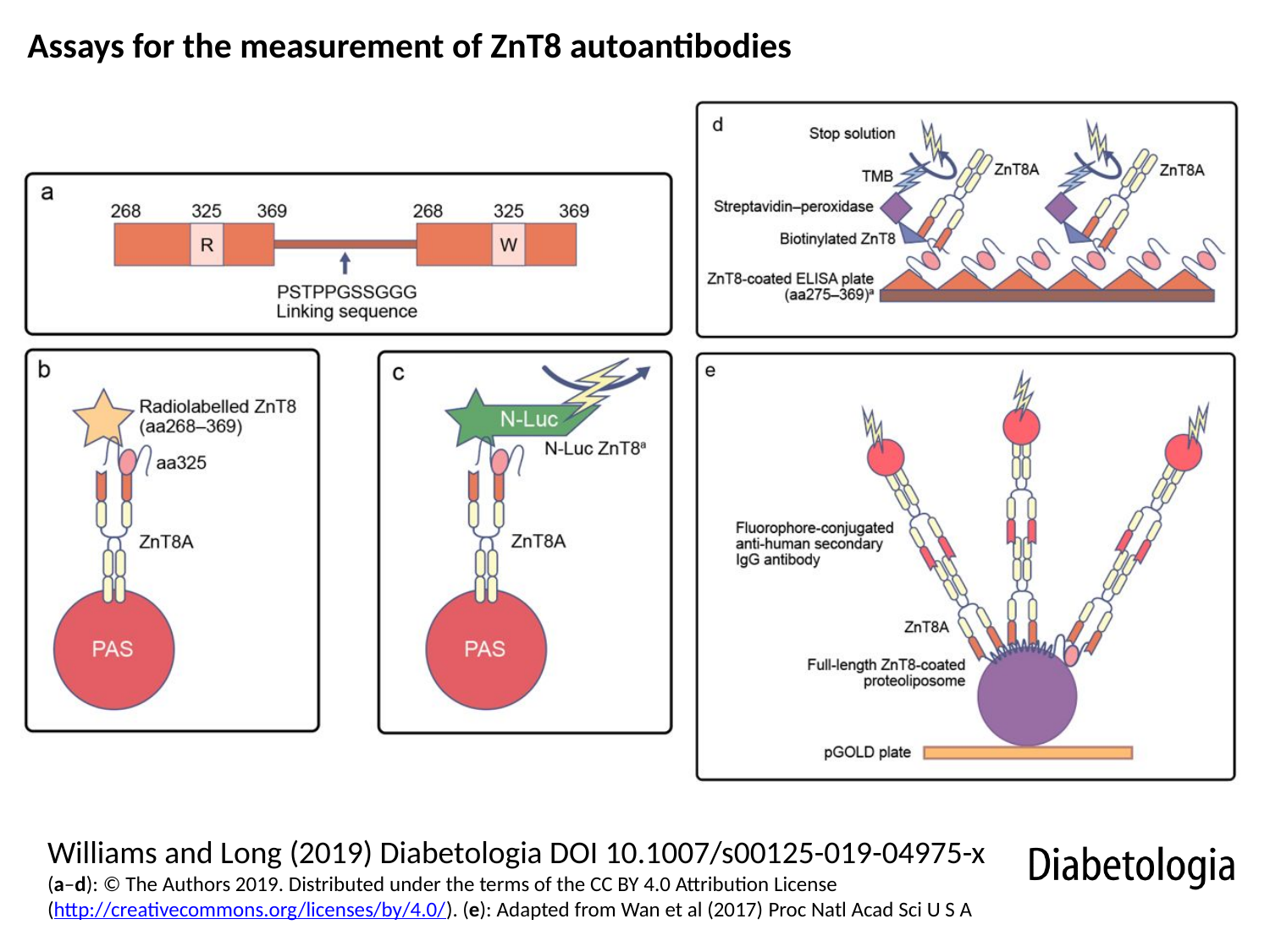

Assays for the measurement of ZnT8 autoantibodies
Williams and Long (2019) Diabetologia DOI 10.1007/s00125-019-04975-x
(a–d): © The Authors 2019. Distributed under the terms of the CC BY 4.0 Attribution License
(http://creativecommons.org/licenses/by/4.0/). (e): Adapted from Wan et al (2017) Proc Natl Acad Sci U S A
